# Supplementary material for: Natural Stabilizers and Nanostructured Lipid Carrier Entrapment for Photosensitive Compounds, Curcumin and Capsaicin
Source: Pharmaceutics. 2024 Mar 17;16(3):412. doi: 10.3390/pharmaceutics16030412 (PMC10975536; doi:10.3390/pharmaceutics16030412)
Supplement: Supplementary file 1 [file pharmaceutics-16-00412-s001.zip › pharmaceutics-2898655-supplementary.pdf]

Supplementary Materials:

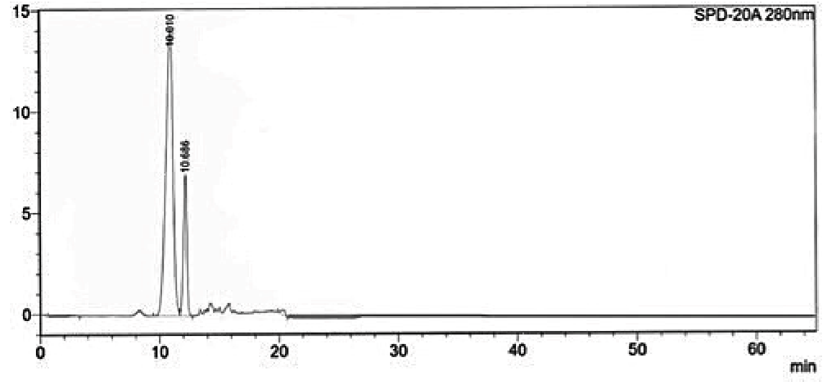

(a) Capsaicin

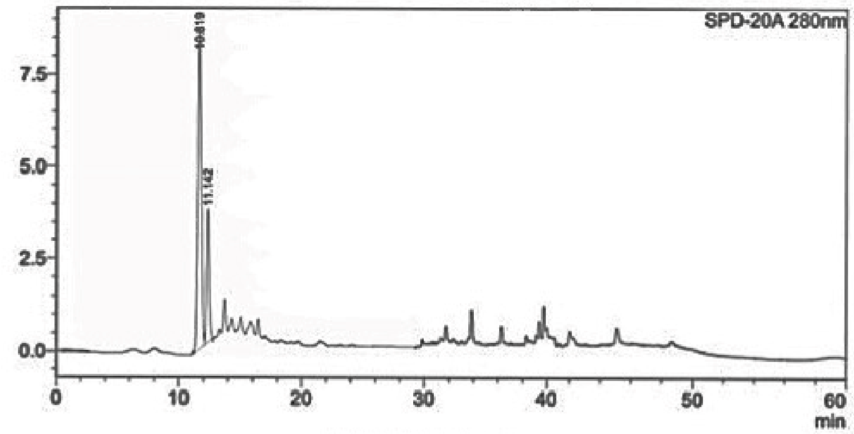

(b) Chili extract

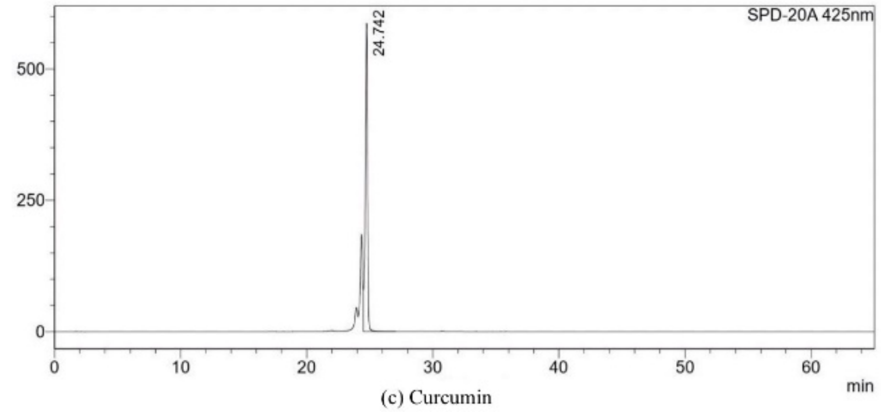

(c) Curcumin

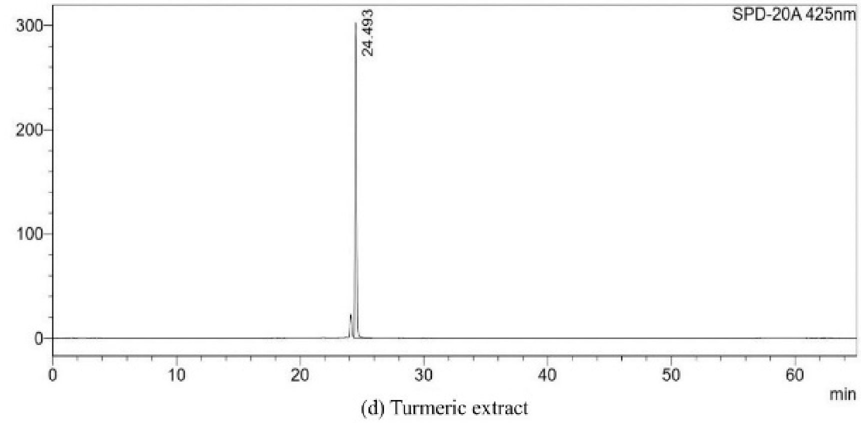

(d) Turmeric extract

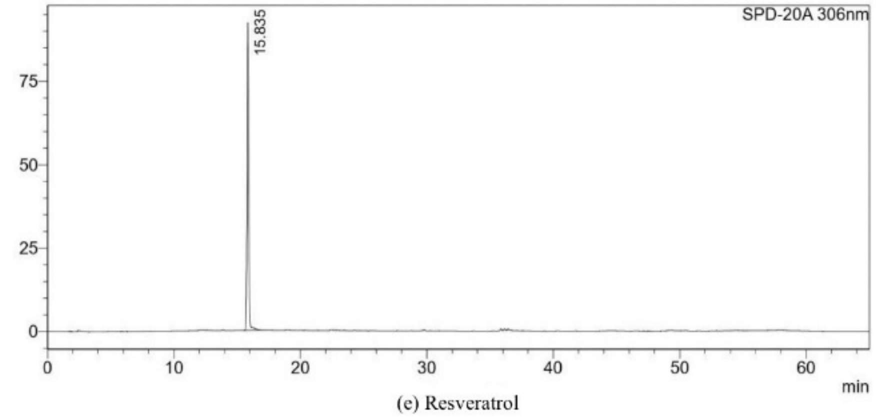

(e) Resveratrol

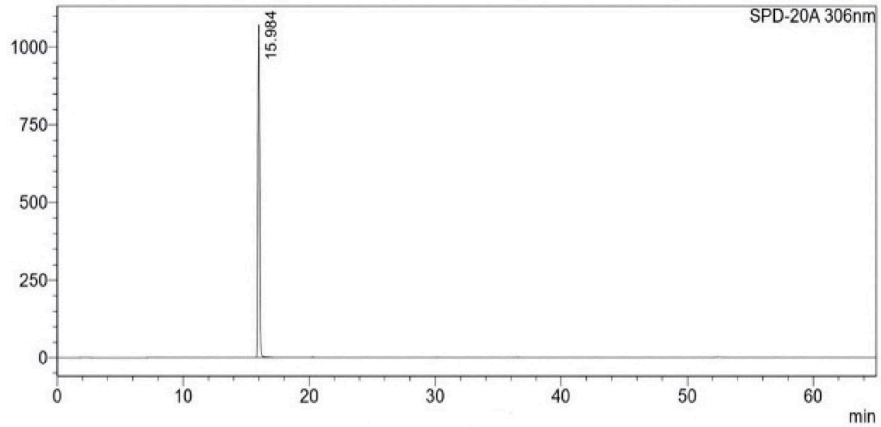

(f) Grape seed extract

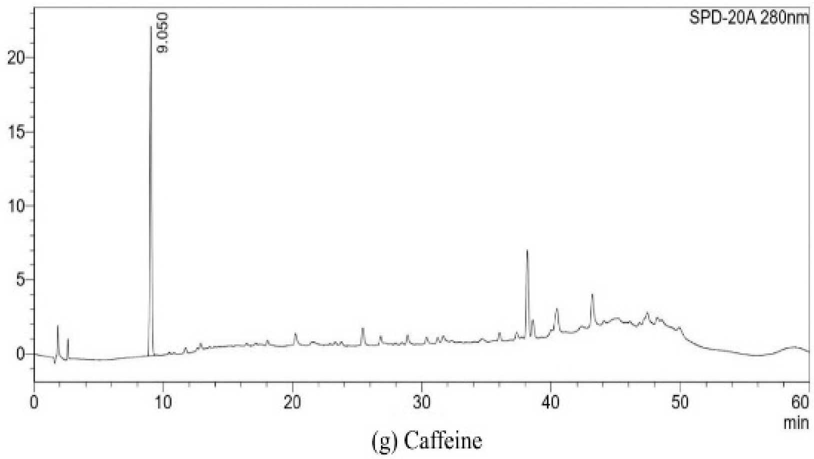

(g) Caffeine

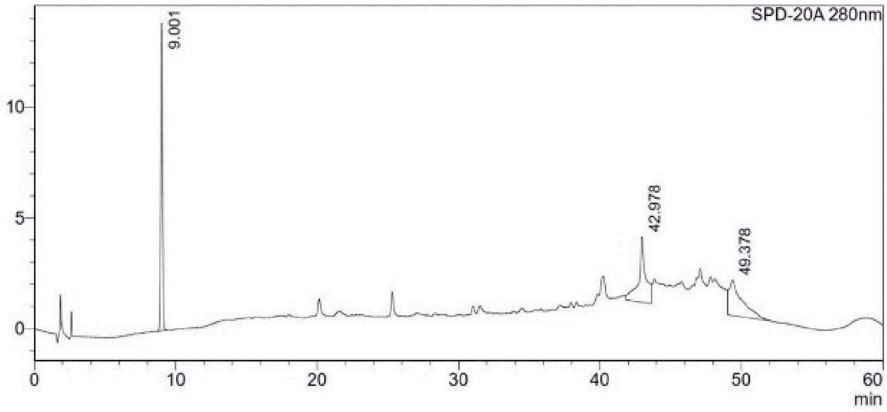

(h) Tea extract

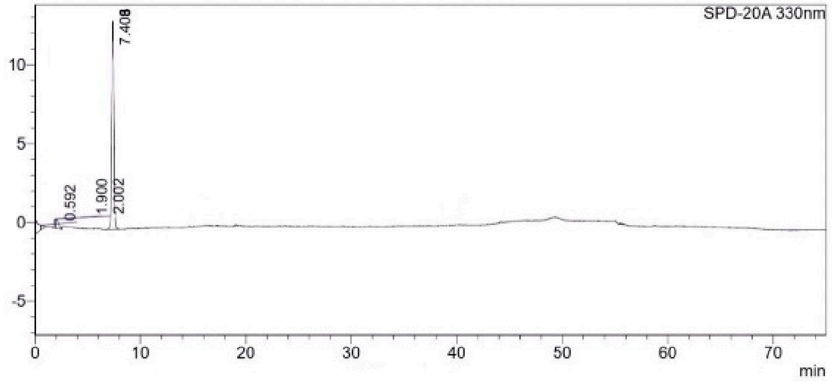

(i) Chlorogenic acid Standard

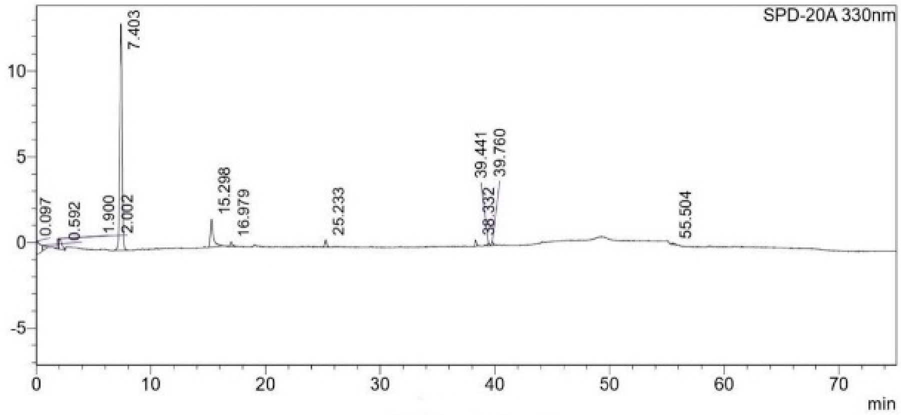

(j) Chlorogenic acid

Figure S1. HPLC chromatograms of capsaicin (a), chili extract (b), curcumin (c), turmeric extract (d), resveratrol (e), grape seed extract (f), caffeine (g), tea extract (h), chlorogenic acid standard (i) and chlorogenic acid (j).

Table S1 Solubility of extracts in natural oils.

**Extract/Compound**	**Oil**	**Volume (mL) of solvent that can dissolve 0.0010 g of solute**	**Solubility**
Turmeric	Sunflower oil	1	Freely soluble
Chili		1	Freely soluble
Tea		5	Sparingly soluble
Chlorogenic acid		1	Freely soluble
Grape seed		1	Freely soluble
Turmeric		1	Freely soluble

Chili	Olive oil	1	Freely soluble
Tea		5	Sparingly soluble
Chlorogenic acid		2	Soluble
Grape seed		1	Freely soluble
Turmeric	Coconut oil	5	Sparingly soluble
Chili		5	Sparingly soluble
Tea		5	Sparingly soluble
Chlorogenic acid		5	Sparingly soluble
Grape seed		1	Freely soluble

Table S2 Characterizations of extract mixtures loaded NLCs.

Extract mixtures loaded NLCs	Mean ± SD		
	Size (nm)	PDI	Zeta potential (mV)
Day 0	153.73 ± 1.97	0.25 ± 0.00	-104.33 ± 2.52
Day 30	152.63 ± 2.41	0.21 ± 0.00	-60.27 ± 1.56*

Asterisk (*) is significantly different at $p < 0.05$ when compared between day 0 and Day 30 that calculated by Paired sample T-test by the SPSS program.

Table S3 Percent entrapment efficiency and percent loading capacity (%LC) of extract mixtures loaded NLCs**.**

Compound	%EE ± SD	%LC ± SD
Capsaicin	95.94 ± 0.05	0.56 ± 0.00
Curcumin	97.79 ± 0.36	0.29 ± 0.00
